# Formaldehyde and De/Methylation in Age-Related Cognitive Impairment

**DOI:** 10.3390/genes12060913

**Published:** 2021-06-13

**Authors:** Ting Li, Yan Wei, Meihua Qu, Lixian Mou, Junye Miao, Mengqi Xi, Ying Liu, Rongqiao He

**Affiliations:** 1 Bayannur Hospital, Bayannur 015000, China; liaishengwu@163.com; 2 State Key Laboratory of Brain and Cognitive Science, Institute of Biophysics, Chinese Academy of Sciences, 15 Datun Road, Chaoyang District, Beijing 100101, China; yanwei@ibp.ac.cn (Y.W.); moulixian@163.com (L.M.); hanfenger86@163.com (J.M.); 3 Translational Medical Center, Weifang Second People’s Hospital, The Second Affiliated Hospital of Weifang Medical University, Weifang 261041, China; qumeihua2016@163.com; 4 School of Life Sciences, Beijing University of Chinese Medicine, Beijing 102488, China; ximengqi7@163.com (M.X.); yingliu69@163.com (Y.L.)

**Keywords:** formaldehyde, epigenetics, age-related cognitive impairment, methylation, demethylation, genotoxin, Alzheimer’s disease

## Abstract

Formaldehyde (FA) is a highly reactive substance that is ubiquitous in the environment and is usually considered as a pollutant. In the human body, FA is a product of various metabolic pathways and participates in one-carbon cycle, which provides carbon for the synthesis and modification of bio-compounds, such as DNA, RNA, and amino acids. Endogenous FA plays a role in epigenetic regulation, especially in the methylation and demethylation of DNA, histones, and RNA. Recently, epigenetic alterations associated with FA dysmetabolism have been considered as one of the important features in age-related cognitive impairment (ARCI), suggesting the potential of using FA as a diagnostic biomarker of ARCI. Notably, FA plays multifaceted roles, and, at certain concentrations, it promotes cell proliferation, enhances memory formation, and elongates life span, effects that could also be involved in the aetiology of ARCI. Further investigation of and the regulation of the epigenetics landscape may provide new insights about the aetiology of ARCI and provide novel therapeutic targets.

## 1. Introduction

Due to the longevity of the human population, aging and aging-related problems have become one of the most important health problems in the world. Age-related cognitive impairment (ARCI) is a predominant syndrome of aging that has attracted increasing attention in the past few decades. Although numerous hypotheses about the mechanisms and risk factors associated with ARCI development have been suggested, only a few effective therapies are clinically available [1]. One of the potential factors contributing to ARCI is dysmetabolism of formaldehyde (FA). FA has also been suggested to be implicated in the development of Alzheimer’s disease (AD), since elevated FA levels in AD patients and animal models of AD are associated with impaired cognitive abilities. In 1999, Luo et al. investigated the effect of aldehydes, such as acetaldehyde and glutaraldehyde on Tau phosphorylation and aggregation [2]. In 2001, Yu hypothesised that cerebrovascular semicarbazide-sensitive amine oxidase is involved in the pathogenesis of Alzheimer’s disease (AD) and vascular dementia [3]. Later, FA was found to cause protein Tau dysfunction and aggregation, leading to cytotoxicity [4,5,6]. Moreover, clinical investigations have shown that dysmetabolism of endogenous FA may be a risk factor for the development of ARCI [7]. Further investigation has contributed to elucidate the pathological relationship between the dysmetabolism of FA and ARCI, as well as the early clinical symptoms of AD [8,9,10,11].

The release of FA by drug demethylation was first described by Axelrod in 1956 [12]. In 1989, DNA methylation was considered a molecular mechanism of cell memory [13,14]. The one carbon cycle is an essential metabolic hub in the cell that releases FA [15]. In 1999, Tohji et al. suggested an association between DNA methylation and neurodegeneration, postulating that the decrease in methylcytosine in the region comprised between base pairs positions −224 to −101 of the amyloid precursor protein (APP) gene promoter in human cerebral cortex autopsy samples was associated with age [16]. Afterwards, it was discovered that the methylation status of cytosines in the *TAU* gene promoter region changes with aging, resulting in a downregulation of the transcriptional activity of this gene in the human brain [16]. Tong et al. further elucidated the effect of FA on epigenetic changes associated with ARCI, and reported a relationship between FA and memory, as well as between FA and the extent of DNA and histone methylation [17]. Later, they identified a relationship between memory loss and FA due to an imbalance in DNA methylation and demethylation [18]. Furthermore, they also determined that high concentrations of FA related to age interfere with the function of DNA methyltransferase, leading to memory loss in AD [19]. In 2017, the relationship between FA and cognition was systematically investigated and elucidated, including the mechanisms of FA synthesis, metabolism, and its role in cognitive impairment, which involved epigenetic mechanisms [20]. Epigenetic alterations and the involvement of FA in memory loss suggest that the exploration of FA exposures as a risk factor may provide an insight into the pathological mechanisms of AD [21]. Here, we discuss the role of FA in epigenetics and its involvement in the development of ARCI through different mechanisms.

## 2. Formaldehyde

As the simplest carbonyl compound, FA has two hydrogen atoms, one oxygen atom, and a carbon atom in the middle (CH_2_O). However, unlike other simple carbon compounds, FA is reactive and can exist in several forms, such as monomeric FA, trioxane, and paraformaldehyde [22]. FA in the upper atmosphere contributes approximately to 90% of the total environmental FA. Atmospheric FA is produced through the action of sunlight and oxygen on atmospheric methane and other hydrocarbons, after which it becomes part of the smog. In forest fires, car exhaust, and tobacco smoke, FA is an intermediate product in the oxidation of methane, and can be oxidized to formic acid [23]. Although FA is colorless, pungent, and toxic, it is an important precursor of many materials and chemical compounds, mainly used in particle boards and coatings, as well as in other industries. FA poses a significant danger to human health, since it presents toxic [24] and carcinogenic characteristics [25]. Ingestion or inhalation of FA has been reported to be harmful to humans, causing eye irritation, headache, difficult breathing, leukemia, cancer, or even death [26,27]. Thus, the use of FA is highly regulated in numerous countries worldwide.

In mammalian organisms, FA is ubiquitous and is an essential intermediate in cellular metabolism. Humans quickly convert FA into formic acid, inhibiting the accumulation of FA in the body [28]. However, humans metabolize FA slower than rodents. The concentration of FA in the blood of human is ~0.1 mM [29]. Endogenous FA refers to that produced in various metabolic pathways involving many compounds, such as sugars, lipids, proteins, and nucleic acids [30,31,32]. For example, FA is formed during the metabolism of methanol or other methylated compounds, such as amino acids [33]. The main enzymes that metabolize endogenous FA are mitochondrial aldehyde dehydrogenase 2 (ALDH2) and glutathione (GSH)-dependent alcohol dehydrogenase 3 (ADH3) [34,35,36,37]. In addition, the intestinal microbiota also consume the excess of FA, combining it with tetrahydrofolic acid (THF) through the one-carbon cycle [20]. Furthermore, biomacromolecules, including proteins, DNA, and RNA, react with the accumulated FA [38]. Methylation/demethylation of DNA, RNA, and histones is considered one of the main ways to form and consume FA in vivo, as well as one of the main mechanisms of epigenetic regulation [39,40,41].

## 3. Methylation and Demethylation in DNA, RNA, and Histones

Epigenetic changes refer to chemical modification of specific genes or gene-associated proteins such as histones that influence gene expression [42,43]. Epigenetic modifications can affect the expression levels of the modified genes and play an important role in a wide range of cellular processes, such as cell fate and organism development. Furthermore, epigenetic modifications can also result in damaging effects, such as the promotion of tumorigenesis. Unlike genetic changes, which are irreversible alternations of the DNA sequence, epigenetic changes are reversible, and, hence, can be targeted by drugs to treat of diseases associated with epigenetic imbalance. Epigenetic changes are generally divided into three classes: DNA methylation, histone modification, and RNA modification. All of these changes lead to alterations in chromatin that affect gene expression through different mechanisms [44]. The folate-driven one-carboncycle, involving the release and usage of FA, is a fundamental metabolic hub in cells that enables the synthesis of amino acids and nucleotides, as well as epigenetic modifications [38].

### 3.1. DNA Methylation

DNA methylation on cytosine bases at cytosine-guanine dinucleotide (CpG) sites is the most common epigenetic mechanism [45,46]. The addition of a methyl group converts cytosine to 5-methylcytosine (5mC). The term CpG islands is used to refer to regions rich in GC nucleotides encompassing hundreds of base pairs, and are commonly located in the promoter region of a gene. Methylation usually plays a negative role in the regulation of gene expression, inhibiting it. In addition, non-CpG sites, such as CpA, CpT, and CpC, can also be methylated to control gene expression [47].

DNA methyltransferases (DNMTs), including DNMT1, DNMT3a, and DNMT3b, are responsible for cytosine methylation [48]. The methyl groups are often obtained from intermediaries of the one-carbon metabolic pathway, such as FA, which can be utilized to form 5-hydroxymethylcytosine (5hmC). FA can then be released from 5hmC, which is then reconverted to an unmodified C in a reverse reaction by the catalysis of DNMTs [49]. During DNA replication, cytosine methylation is maintained and transmitted to the daughter cells. CpGs are hemimethylated during DNA replication. The cytosine methylation pattern in the new DNA strand is maintained by DNMT1, which is a component of the DNA replication complex. DNMT3a and DNMT3b are mainly expressed in the early developmental stages and are responsible for de novo DNA methylation. Their association with DNMT3L, which recognizes unmethylated histone H3 lysine 4 (H3K4), is required for DNA methylation. *DNMT* genes are crucial for development, and deletion of *DNMT* genes can lead to early embryonic lethality [50,51]. Drugs, such as 5-azacitidine, are known to degrade DNMTs and block DNA methylation [52,53].

DNA demethylation relies on enzymes of the ten-eleven translocation (TET) family of methylcytosine hydroxylases, including TET1, TET2, and TET3 [54,55]. The hydroxylase activity of these enzymes converts 5mC to 5hmC, 5-formylcytosine (5fC), and finally to 5-carboxylcytosine (5caC). Further decarboxylation of 5caC leads to its conversion into cytosine. The roles of the intermediates of DNA demethylation in epigenetic control are still under investigation [56].

DNA methylation results in gene silencing through two main pathways: first, it can block transcription factors or regulators directly by affecting the recognition sites; second, regulation through methyl-CpG binding protein 2 (MeCP2), which can recruit histone acetyl deacetylases (HDACs) that remove gene activation-associated markers. In addition, oxidative DNA demethylation corrects the toxic lesions 1-methyladenine and 3-methylcytosine, catalyzed by DNA dioxygenases. These enzymes release the methyl moiety, directly reversing the base alteration [57]. Defects in DNA methylation have been reported in several diseases, such as cancer [58].

### 3.2. Histone Modification in Lysine Specific Demethylase (LSD) Releases Two Molecules of Formaldehyde

Histones are a highly conserved family of proteins that present a similar structure, consisting of a globular domain and a flexible amino terminal tail. Several amino acid residues in the globular and tail domains can be modified by post-translational modifications, which impacts histone activity and, therefore, contribute to chromatin variability. FA can participate in histone modification acting as a methyl group donor. The alteration of the chromatin structure can facilitate or impede gene transcription. Several histone posttranslational modifications have been well characterized, including methylation, acetylation, and phosphorylation. Among them, methylation is one of the most thoroughly investigated modifications [59,60].

Lysine and arginine residues in the histone terminal tails are the residues that are most commonly modified, and can be either methylated by methyltransferases, or demethylated by demethylases [61]. The epigenetic effects of histone methylation depend on the location of methylated histone and the amount of methyl groups on it, either promoting or suppressing transcription [62]. For example, in histone H3, one of the most extensively studied histones, some types of methylation, such as di-methylation at lysine 4 (H3K4me2) and mono-methylation at lysine 9 (H3K9me1), result in active transcription. However, di- and tri-methylation of histone H3K9 (H3K9me2 and H3K9me3) cause transcription repression. Lysine-specific histone demethylase 1 (KDM1A or LSD1), a member of the flavin adenine dinucleotide-dependent amine oxidase family, can demethylate mono- and di-methylated H3K4 and H3K9 residues. Shi et al. determined whether FA was produced in LSD1-mediated enzymatic reactions using a formaldehyde dehydrogenase (FDH) assay [63].

### 3.3. RNA Modification

Although DNA methylation and histone modification are important epigenetic changes, RNA modifications should not be overlooked. The first discovered and the most abundant modification of messenger RNA (mRNA) and long non-coding RNA (lncRNA) is N6-methyladenosine (m6A) [64]. Over three sites were modified for every mRNA molecule. Other common RNA modifications include pseudouridine, 5mC, 7-methylguanine, N1-methyladenosine, and 2-O-Methyl.

m6A, which refers to the methylation of N6 of adenosine, often occurs in the consensus sequence RRACH (R = G or A; H = A, C, or U), and is mediated by m6A methyltransferases [64]. In 2011, the methylation of mRNA was demonstrated to play a critical role in human energy homeostasis. Furthermore, the identification of fat mass and obesity-associated (*FTO*) gene, encoding the first RNA demethylase discovered, indicates that RNA modification can be an epigenetic marker [65]. FTO is a member of the α-ketoglutarate and Fe^2+^-dependent AlkB family that catalyzes the oxidative demethylation of DNA and RNA bases [66]. FTO is associated with diabetes and obesity in humans [67,68], and recently it has been reported to be also associated with cellular processes, such as adipogenesis, regulation of dopaminergic signaling, and mRNA splicing [69]. Demethylation of m6A by ALKBH5 not only results in the release of FA, but also regulates the release rate of FA [70]. In addition to FTO, many other enzymes are involved in m6A modification, like methyltransferases, demethylases, or regulators of m6A, such as MTTTL3, ALKBH5, and YTHDF1 [71]. Enzymes involved in m6A modification are classified as ‘writers’ that catalyze chemical modification at specific sites, ‘erasers’ that remove modifications, and ‘readers’ that recognize the modified sites in DNA or histone [72]. To date, m6A modification is the only RNA modification for which enzymes involved in these three distinct steps have been discovered. Although m6A modification of mRNA and lncRNA has been linked to human health and disease, limited knowledge is available about other modifications of mRNA and lncRNA, highlighting the need for deeper investigation of RNA epigenetics.

Besides lncRNA, other non-coding RNAs (ncRNAs) play important roles in epigenetic and gene expression regulation, such as small-interfering RNAs (siRNAs), which are 19–24 bp-long RNA molecules derived from long double-stranded RNA; microRNAs (miRNAs), which are ~19–24 nt single-stranded RNAs; and piwi-interacting RNAs (piRNAs), which are ~26–31 nt RNA molecules [73,74]. These RNAs exert their regulatory functions mainly through the interaction with DNA, scaffolding proteins involved in chromatin modification and remodeling, or through the recruitment of transcription regulating factors [75]. FA is also involved in the regulation of ncRNA, but the de/methylation of ncRNAs themselves remains to be elucidated.

## 4. Formaldehyde in Epigenetics

FA serves as a methyl donor in the one-carbon cycle, making FA an epigenetic factor that plays a role in DNA, RNA, and histone methylation. DNMT employs S-adenosyl-L-methionine (SAM) as the methyl donor in the methylation of DNA/RNA/histone through the ‘one-carbon pool’ in humans. Furthermore, the methyl group of 5,10-Methylene THF, the product of the reaction of FA with THF, is necessary for the formation of methionine. Hence, FA is a product of DNA demethylation, and its derivative 5,10-methylene-THF is a methyl donor in DNA methylation [76]. Generation of endogenous FA often follows the absence of methylation maintenance after replication, followed by the demethylation of deoxycytidine and 5-azacytidine. Active demethylation, which is not related to DNA replication, is also accompanied by FA production. Demethylation occurs in the oxidative methyl group of cytosine and is catalyzed by glucosyltransferase, releasing cytosine and endogenous FA.

Similar to DNA demethylation, RNA demethylation also results in the production of FA. Oxidation of m6A at the methyl group produces N6-hydroxymethyladenosine (hm6A), which can undergo further oxidation producing N6-formyladenosine (f6A), FA, and formic acid. FA, SAM, and histones are involved in reversible histone methylation. Methylation of lysine and arginine residues of histone (H3/H4) depends on the methyl group of SAM supported by FA [77], but the metabolism of methylated histone residues also results in the production of FA [62]. The demethylation of mono-methylated H3K4 catalyzed by LSD1 results in the production of two molecules of FA. Furthermore, the reduction of the cofactor FAD to FADH2, and the subsequent oxidation of FADH2 results in the production of hydrogen peroxide and FA in a complete catalytic cycle. Moreover, semicarbazide-sensitive amine oxidase catalyzes methylamine to produce FA in another metabolic pathway [78].

Endogenous FAs participate in the reversible methylation of DNA, RNA, and histones, which are involved in the regulation of epigenetic memory in chromosome segregation, transcriptional regulation, and DNA repair [76]. Moreover, exogenous FA has also been reported to induce epigenetic alterations similar to those induced by chemical carcinogens. Exposure to FA in industrial installations, from tobacco smoke, automobile exhaust, and other sources leads to devastating consequences, such as cancer and leukemia due to the reaction of FA with DNA and proteins and the involvement of FA in epigenetic changes. Global loss of DNA methylation is an epigenetic alteration associated with genomic instability and has been observed in FA-treated human 16HBE cells [79]. Furthermore, FA exposure also leads to downregulation of DNMT3a and DNMT3b, resulting in the hyperphosphorylation of histone H3 in human fibroblasts [80]. In addition, the balance between histone acetylation and deacetylation can be disturbed by binding of FA to lysine residues [81]. Finally, dysregulation of miRNAs by FA exposure has been reported to be involved in inflammatory responses, apoptosis, and several human diseases [82,83].

### Double-Edged Effects of Formaldehyde

**Figure 1:**
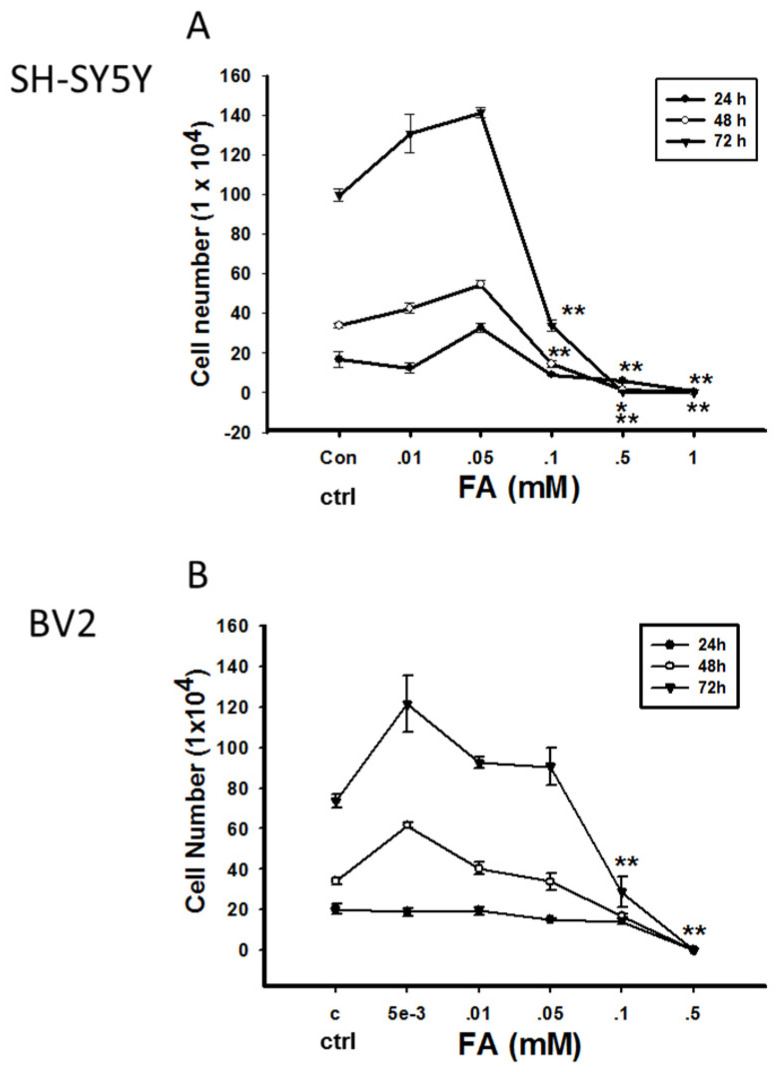
Effect of exogenous formaldehyde (FA) on the viability of SH-SY5Y cells and BV2 cells. SH-SY5Y cells and BV-2 cells were cultured with different concentrations of FA in Dulbecco’s Modified Eagle Medium containing 10% fetal calf serum (37 °C, 5% CO_2_). The viability of cells was assayed using the CCK8 assay at different time points. Cell viability of SH-SY5Y (**A**) and BV-2 cells (**B**) is shown, respectively. Cells without FA treatment were used as control (*n* = 3; means ± S.E.M; * *p* < 0.05, ** *p* < 0.01).

**Figure 2:**
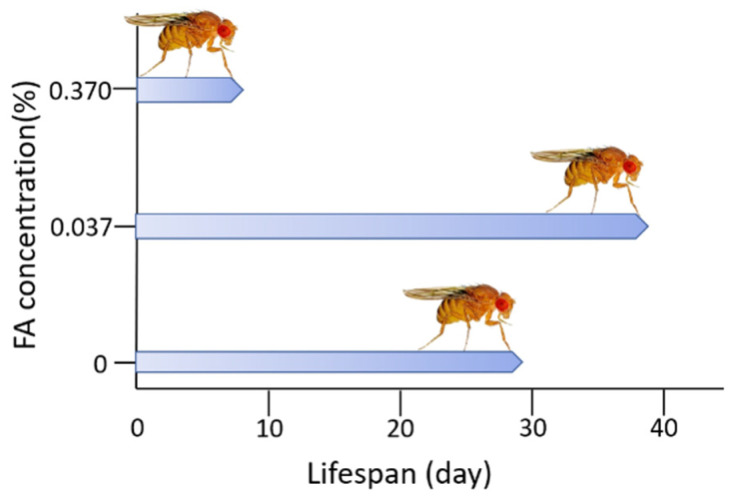
Feeding with a low concentration of formaldehyde (FA) extends average life span of female Drosophila. The lifespan of Drosophila was extended by feeding 0.037% FA and shortened by 0.370% FA, adopted from Li and He [87].

Although FA exerts toxic activity, especially called genotoxicity, it is also indispensable for cellular metabolism under physiological conditions. Whether endogenous FA plays a beneficial or detrimental role in cells depends on its concentration. Incubation of neural cells with different concentrations of FA for three days resulted in an increase in cell proliferation when FA was administered at lower concentrations (0.001–0.05 mM for BV2 cells; 0.01–0.05 mM for SH-SY5Y cells), whereas treatment with higher FA concentrations (> 0.05 mM) resulted in increased cell death. Similar results were reported for N2A cells, which demonstrated an increase in viability in the presence of a low concentration of FA (0.01 mM), but continuous daily addition of FA reduced the cell number (Figure 1) [84]. In addition, Tong et al. described that normal adult rats intrahippocampally injected with FA present spatial memory deficits and global loss of DNA methylation similar to those associated with age. However, both scavenging elevated FA by FDH administration or enhancing DNA demethylation by a DNA demethylating agent caused spatial memory deficits in rats [18]. Furthermore, it has been observed that excessive FA in the brain impairs cognitive abilities such as learning and memory. However, overexpression of ADH5, which acts as a scavenger of FA, leads to cognitive impairment in *Drosophila,* although FDH is associated with the NO metabolic pathway [85,86]. The multifaceted effect of FA in relation with aging have also been elucidated in several animal models. In *Drosophila*, different concentrations of FA have been found to play different roles in lifespan and stress resistance (Figure 2) [87].

## 5. Formaldehyde-Involved Epigenetics in Age-Related Cognitive Impairment

ARCI is characterized by problems with memory, language, thinking or judgement, and it is a typical feature of AD. Most cases of AD are sporadic, and numerous factors have been identified as risk factors, including aging, family history, diabetes, obesity, hypertension, and diet [88]. Although the pathological mechanism of AD remains to be elucidated, two main hypotheses remain: ‘accumulation of amyloid-β’ and ‘tau pathology’. Besides these, numerous other pathways are known to be involved in the aetiology of AD, such as oxidative stress, inflammation, calcium channel disruption, and autophagy [89]. In familial AD, mutations in *APP*, *PSEN1* and *PSEN2* are known genetic risk factors [90].

**Figure 3:**
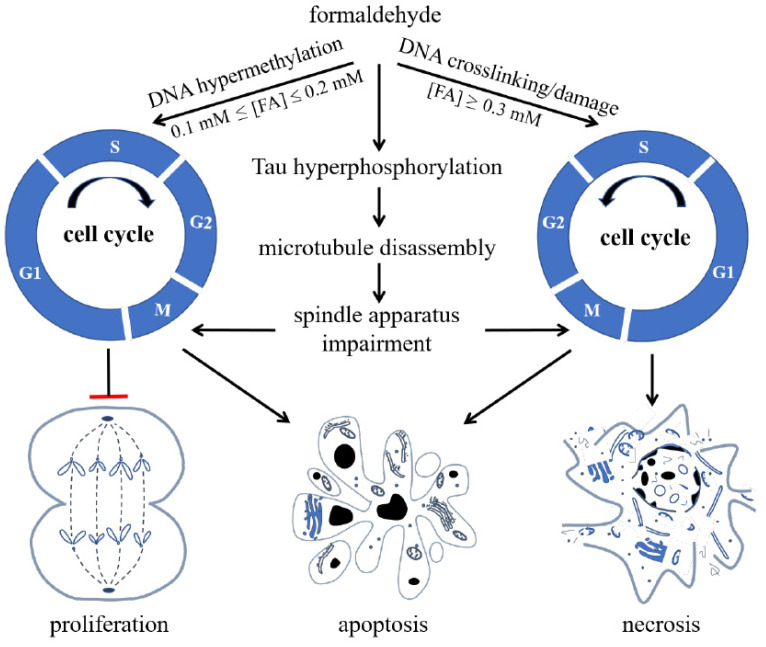
Formaldehyde (FA) inhibits cell proliferation and induces cell apoptosis and necrosis via its effect on cell cycle. FA induces hypermethylation of DNA when 0.1 mmol/L ≤ (FA) ≤ 0.2 mmol/L, and causes crosslinking or damage of DNA, arresting cells at S phase and restraining cells from entering G2/M phase when (FA) ≥ 0.3 mmol/L, reproduced with permission from Miao et al. [9].

Bohnuud et al. reported that FA can cause hydroxymethylation of the pyrimidine bases in DNA and transform them into methyl groups through oxidation, silencing gene expression [91]. Furthermore, Lu et al. found that FA can react with DNA, resulting in methylated DNA [92], which can silence gene expression and inhibit DNA synthesis in cells [93,94]. When the FA concentration increases, it acts on nuclear DNA, aggravates the degree of DNA methylation, and then inhibits DNA unchaining, replication, and synthesis. Hence, high concentrations of FA cause cell cycle arrest, eventually triggering apoptosis and necrosis (Figure 3) [95].

ARCI originates from chronic synaptic dysfunction [96] and neuronal loss [97], which are accompanied by the formation of senile plaques, which are extracellular deposits of amyloid-β (Aβ), and neurofibrillary tangles, which are paired helical filaments of hyperphosphorylated Tau. Excessive FA induces hyperphosphorylation of Tau, resulting in microtubule disassembly and damage of the spindle apparatus. In SH-SY5Y cells, these effects cause cell cycle arrest at the G2/M phase. Excessive FA also has an aberrant impact on DNA [98]. Animal experiments have shown that methanol, which is converted to FA in vivo, can induce amyloid β deposition, Tau hyperphosphorylation, and cognitive impairment in monkeys [99,100]. In patients with AD, the concentration of Aβ in the CSF is decreased, indicating an increase in Aβ deposition in brain tissue [101]. In *Rhesus* monkeys, FA concentration in CSF increases with aging and is negatively correlated with Aβ concentration [102], further supporting a link between FA concentrations and Aβ accumulation.

Fei et al. reported oxidative demethylation at Ser8/26 of Aβ induces FA generation. According to their results, FA interacts with the Lys28 in the β-turn of Aβ monomers, enhancing Aβ oligomerization [103]. Furthermore, Aβ inhibits FDH activity, leading to FA accumulation. Therefore, excessive FA and Aβ oligomers form a positive feedback cycle that may play a role in the development of ARCI. FA scavengers such as resveratrol, NaHSO_3_, or coenzyme Q10 have been reported to reduce Aβ aggregation, ameliorate neurotoxicity, and improve cognitive performance in APP/PS1 mice [10,103]. The direct interaction between FA and Tau protein methylation is yet to be investigated.

These results suggest that FA may be a risk factor for sporadic ARCI, although it is not present in all patients with AD. Endogenous FA is closely related to ARCI in patients with AD [7]. High endogenous FA levels have been reported in patients with AD in comparison with age-matched participants with normal cognition [104]. Moreover, exogenous FA exposure have been shown to induce AD-like changes in murine brain [105]. Endogenous levels of FA could be employed as a diagnostic criterion for ARCI in the preclinical and clinical stages, and for epidemiological investigation of AD. The mechanisms involved in the dysmetabolism of FA in ARCI include FA-induced amyloid peptide aggregation, tau pathology, inflammation, oxidative stress, neurotoxicity, and gut microbiota imbalance. However, methylation or demethylation of DNA, RNA, histone, and amino acids are typical features of epigenetic regulation of the disease.

Recent studies have indicated that epigenetics contribute to the development of ARCI. Altered DNA methylation, histone modifications, and ncRNA interactions are characteristics commonly found in AD. According to the epigenetic clock theory of aging, epigenetic changes, including DNA methylation/demethylation, are involved in aging [106,107,108]. In patients with AD, global DNA methylation in brain samples is decreased. Moreover, an altered DNA methylation landscape in CpG islands of susceptible genes in AD, such as *ABCA7* and *BIN1,* have been shown to be associated with AD pathology [109]. Lower levels of 5mC and 5hmC have also been reported using in vitro models of AD and in brain tissue of patients with AD [110]. In addition, alterations in the gene expression level of HDACs have been linked to AD [111]. Excessive histone methylation has also been reported in patients with AD [112]. Finally, ncRNAs, including miRNAs and lncRNAs, were also found to be differentially expressed in AD pathology [113,114].

### Imbalance of Formaldehyde Metabolism Is Associated with Age-Related Cognitive Impairment

In physiological conditions, FA is metabolized and homeostasis is maintained [95]. An imbalance in FA metabolism may be related to the development of ARCI [20]. We believe that one of the main causes of cognitive impairment may be excessive production and accumulation of endogenous FAs in the brain. Although either a lack of or excess of FA resulting from an imbalance in metabolism should be harmful to cognitive ability, the lack or low concentration of endogenous FA is not commonly considered as a major risk factor. This viewpoint is based on the following observations: (1) Endogenous FA originates from several metabolic pathways. If less FA is produced in one metabolic pathway, the production of FA can be compensated for by other metabolic pathways [20]. (2) The degradation pathways of FA, involving enzymes such as ADH and ALDH, are less abundant than those of the production pathways [115]. (3) High levels of FA were observed in the digestion contents in the cecum of 7-month-old APP/PS1-transgenic mice. However, a marked increase in FA was not observed in the cecum contents of wildtype 7-month-old C57 mice [116], nor in 24-month-old wildtype 129S2/SvPasCrl mice though their intestinal length extended with aging [117] (Figure S1, Permit Number: SYXK2019-06). This suggests that FA metabolism in vivo may also be related to the intestinal microbe flora. (4) Methylation and demethylation play critical roles in the catecholamine metabolic pathway. FA acts as a methyl donor in the catecholamine pathway through the SAM cycle [118]. Accumulation of FA disturbs the metabolism of catecholamines, leading to norepinephrine depletion and cognitive decline [119]. (5) Exogenous FA is ubiquitous in humans, despite its concentration [120]. This may explain why no drug to increase FA concentrations has been developed for conditions in which FA concentrations are low. Therefore, we emphasize that FA accumulation resulting from the metabolism imbalance is associated with ARCI.

FA metabolism imbalance is associated with ARCI, as FA acts as a methyl group donor, participating in the methylation or demethylation of DNA, RNA, and histones and regulating the epigenetics landscape. Although the effects of FA on DNA methylation/demethylation have been investigated, the implications of FA in ARCI development need to be further investigated. FA levels influence the activity of DNMT and the degree of DNA methylation in the brain [18]. Excessive FA has shown to decrease DNA methylation by disturbing the activities of DNMTs in vivo and in vitro. During spatial learning in Sprague–Dawley rats, there is an initial global DNA demethylation, followed by a re-methylation step associated with the elevation of hippocampal FA. Subsequently, hippocampal FA levels decrease and reach the baseline levels [18]. Excessive FA concentrations could mimic the global DNA methylation decline associated with age-related spatial memory deficits observed in normal adult rats. Furthermore, global DNA methylation levels in the autopsied hippocampus of patients with AD are associated with a marked increase of endogenous FA levels [19]. Thus, the dysregulation of DNMT related to defects in the global levels of DNA methylation could be one of the pathophysiological mechanisms underlying hippocampal-associated spatial memory impairment. According to this hypothesis, in the elderly, the degree of DNA methylation is reduced and endogenous FA accumulates, leading to the development of ARCI in the most severe cases. Hence, targeting FA imbalance might be an effective way to prevent ARCI. Although no approved drugs can be used for this purpose, some chemicals or compounds that have neuroprotective effects, including some Chinese medicinal herbs (e.g., resveratrol), physical methods, and lifestyle interventions are recommended as a treatment for ARCI.

We hypothesize that endogenous FA, including that derived from catecholamine, APP, Aβ peptide, and Tau protein, acts as an epigenetic factor in the formation or loss of memory associated with the methylation and demethylation of DNA, RNA, and histones. FA dysmetabolism has been suggested as a risk factor for ARCI, as this condition is associated with the disturbance of DNA methylation and demethylation. Further investigations on whether FA is associated with methylation and demethylation of RNA, histones and other biomolecules in ARCI should be performed in the future.

## 6. Conclusion

The review depicted the interplay between FA and epigenetics, involved in ARCI. Homeostasis of formaldehyde metabolism is maintained by several metabolic pathways, including the one-carbon cycle, which involves methylation of DNA, RNA, proteins, and amino acids. FA dysmetabolism, which is associated with the disturbance of methylation and demethylation. has been suggested as a risk factor for ARCI.

## Supplementary Materials

The following are available online at https://www.mdpi.com/article/10.3390/genes12060913/s1, Figure S1. Formaldehyde concentration in the intestine of 129S2/SvPasCrl mice at different ages. Formaldehyde concentration in the intestinal canal, including duodenum (A), jejunum (B), ileum (C), cecum (D), colon (E), and the intestinal contents including jejunum (F), ileum (G), cecum (H), and colon (I) of male and female mice, is presented. The Student’s *t*-test was employed to data in panels A−I. Data were showed as mean ± S.E. **: *p* < 0.01; *n* is the number of mice examined.

## Abbreviations

FA	formaldehyde
ARCI	age-related cognitive impairment
AD	Alzheimer’s disease
de/methylation	demethylation and methylation
