# Supplementary figures and images for: Formaldehyde and De/Methylation in Age-Related Cognitive Impairment
Source: Genes (Basel). 2021 Jun 13;12(6):913. doi: 10.3390/genes12060913 (PMC8231798; doi:10.3390/genes12060913)

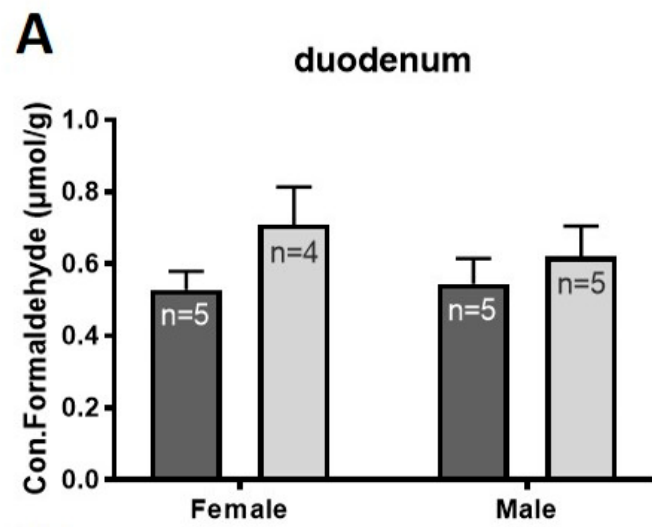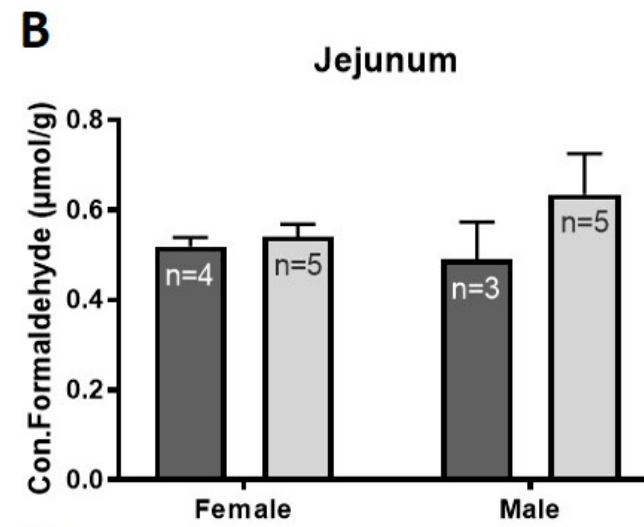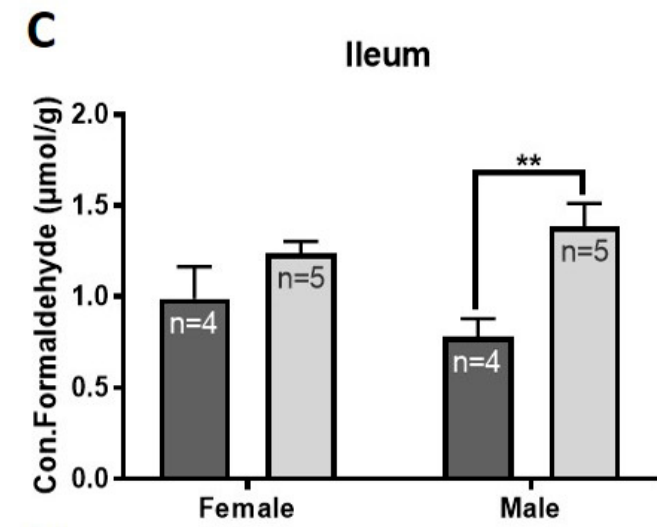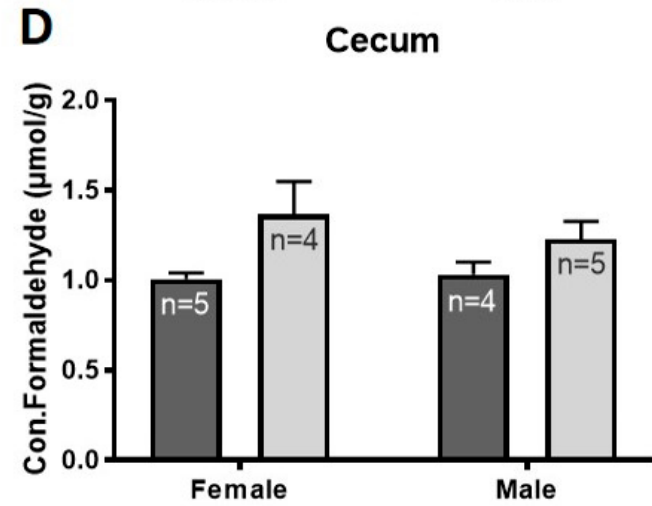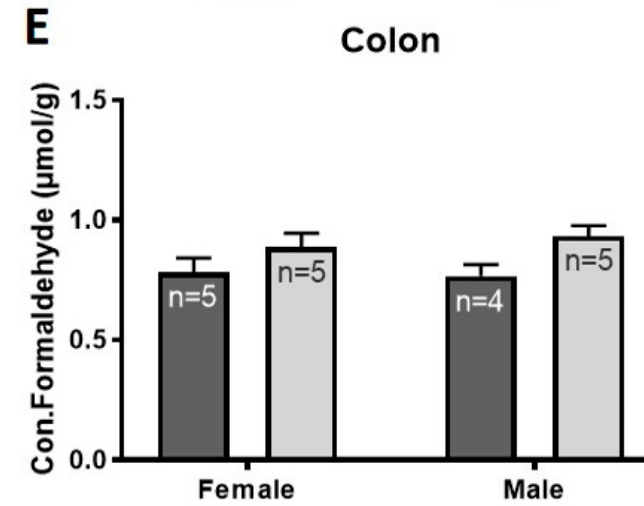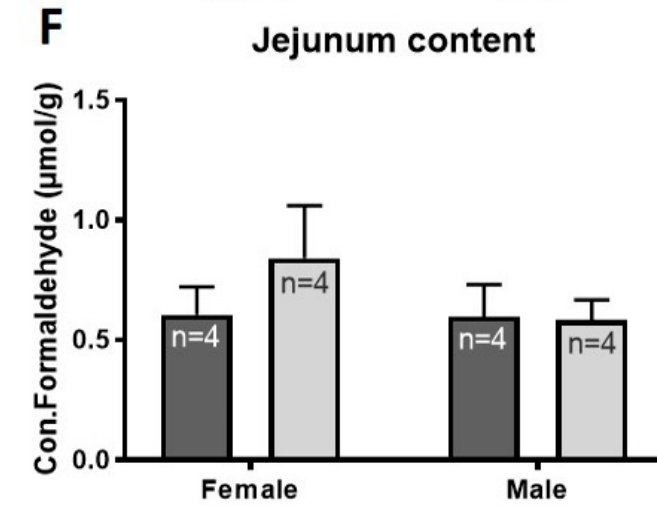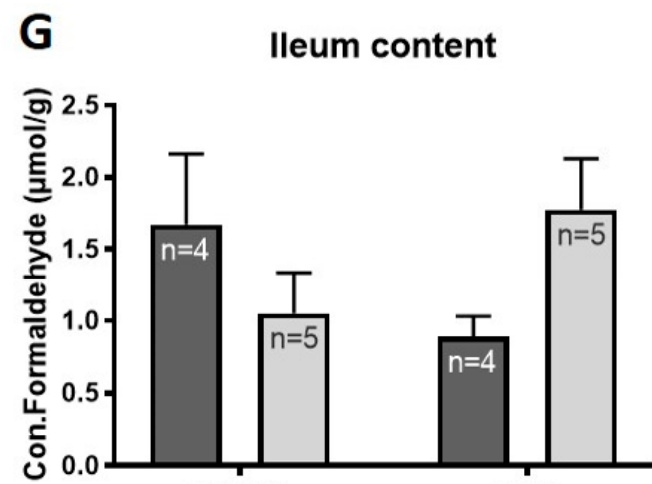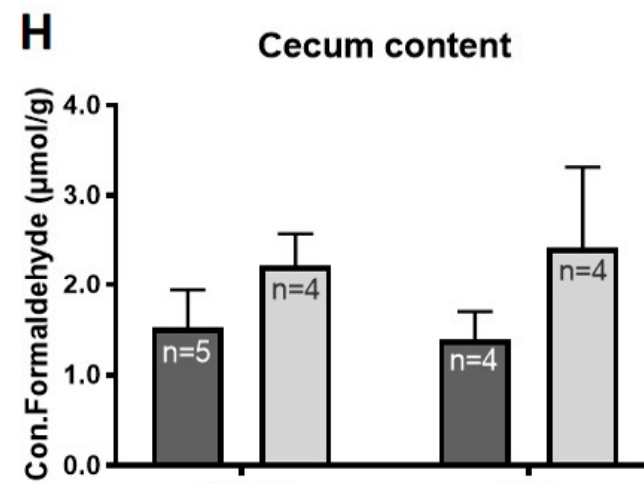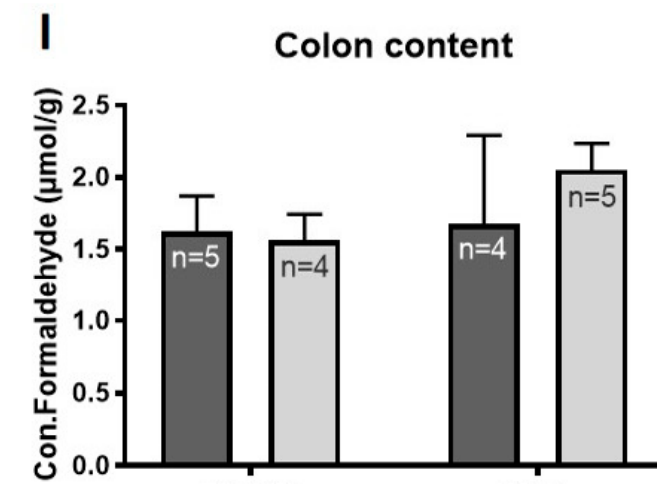

Supplement: Supplementary file 1 [file genes-12-00913-s001.zip › genes-1212410-supplementary.pdf]
